# Pharmacokinetic and Pharmacodynamic Considerations of Antibiotic Use in Neonates

**DOI:** 10.3390/antibiotics12121747

**Published:** 2023-12-18

**Authors:** Mario Regazzi, Alberto Berardi, Simonetta Picone, Chryssoula Tzialla

**Affiliations:** 1 S.I.F.E.B, Italian Society of Pharmacokinetics and Biopharmaceutics, 27100 Pavia, Italy; 2 Neonatal Intensive Care Unit, University Hospital of Modena, 41124 Modena, Italy; alberto.berardi@unimore.it; 3 Neonatology and Neonatal Intensive Care Unit, Policlinico Casilino, 00169 Rome, Italy; simpico@libero.it; 4 Neonatal and Pediatric Unit, Ospedale Civile Voghera, ASST Pavia, 27100 Pavia, Italy; chryssoula.tzialla@unipv.it

**Keywords:** newborns, pharmacokinetics, pharmacodynamics, therapeutic drug monitoring, model-informed precision dosing

## Abstract

The selection of an appropriate dose of a given antibiotic for a neonate not only requires knowledge of the drug’s basic pharmacokinetic (PK) and pharmacodynamic (PD) properties but also the profound effects that organ development might have on the volume of distribution and clearance, both of which may affect the PK/PD of a drug. Interest has grown in alternative antibiotic dosing strategies that are better aligned with the antibiotic’s PK and PD properties. These strategies should be used in conjunction with minimum inhibitory concentration measurements and therapeutic drug monitoring to measure their potential success. They can also guide the clinician in tailoring the delivery of antibiotics to suit an individual patient’s needs. Model-informed precision dosing, such as Bayesian forecasting dosing software (which incorporates PK/PD population models), may be utilized to optimize antibiotic exposure in neonatal populations. Consequently, optimizing the antibiotic dose and exposure in each newborn requires expertise in different fields. It drives the collaboration of physicians together with lab technicians and quantitative clinical pharmacologists.

## 1. Introduction

Establishing the right dose for the drugs used in the treatment of the newborn is of fundamental importance.

Neonates have significant physiological differences affecting the absorption, distribution, metabolism, and elimination of drugs; such differences make it inappropriate to extrapolate dosages from adults and older children [1].

In clinical practice, antibiotics are the most commonly used drugs in newborns, especially those admitted to neonatal intensive care units (NICUs) since sepsis is an important cause of neonatal morbidity and mortality [2,3,4,5,6]. A recent systematic review, a meta-analysis on global incidence and mortality, estimated that 2874 neonates for every 100,000 live births develop sepsis, with a mortality rate of 17.6% [7]. The incidence of sepsis among neonates varies based on birth weight, gestational age, and the type of sepsis (early- or late-onset sepsis). Premature babies present a higher incidence of early-onset sepsis (EOS) compared to full-term infants (15.05/1000 live births vs. 0.5/1000 live births) [8,9] and late-onset sepsis (LOS) compared to EOS (88.5/1000 preterm births vs. 13.5/1000 preterm births) [10,11]. In fact, in 30–35% of term babies, more than 75% of the babies with birth weights are less than 1500 g, and over 80% of those with birth weights of less than 1000 g receive antibiotic therapy [12,13].

A recommended regimen of empiric antibiotic therapy for EOS combines the use of ampicillin and aminoglycosides plus third-generation cephalosporins in cases with suspected meningitis. Regarding LOS, there is no universal recommendation for empiric therapy, but different combinations of antibiotics could be employed based on local microbial resistance profiles, infection source, case severity, and potential risk factors for antibiotic resistance [9,14,15,16]. A survey from 38 European countries found that the most frequently used antibiotics in NICUs for LOS were vancomycin (52.4%), gentamicin (33.9%), cefotaxime (28%), and meropenem (15.5%) [16].

A one-day global point prevalence study in eighty-four NICUs from twenty-nine countries on five continents showed that 26% of hospitalized infants were under antibiotics, and the most prescribed agents for the treatment of presumed/confirmed infections were vancomycin, amikacin, and meropenem [17].

Despite the extent of the use of antibiotics in neonates in quite a few cases, specific dosage information exists. The complexity of prescribing also stems from the need to rapidly adapt the antibiotic dosage to the degree of metabolic immaturity of neonatal organs (liver, kidney, distribution volumes); growth and functional maturation of the physiological processes may affect the pharmacokinetic processes, leading to increased toxicity or reduced efficacy.

The scarcity of available data to define appropriate dosages also results from the difficulty of conducting studies on newborns. This difficulty is due to several factors such as ethical concerns, scarcity of patients suffering from individual diseases that can potentially be enrolled, the objective of the study, a lack of a feasible experimental design, and a lack of experience in the simulation of neonatal pharmacokinetic (PK)/pharmacodynamic (PD) procedures and large-volume blood samples required for pharmacokinetic studies [1,18].

The need for neonatal-specific data is also underlined by the fact that newborns represent a heterogeneous population in which body weight can vary more than 10 times, the gestational age may vary between 24 and 42 weeks, and the postnatal age may range from 0 to 30 days [1].

In recent years, the European Union has favored the adoption of appropriate legislation for the inclusion of children and infants in drug trials; this has led to an increase in pediatric studies, despite organizational and ethical challenges in studying drugs for neonates [19,20].

To address these challenges, new study design strategies have been developed, including the use of risk-minimizing methods in assessing the pharmacokinetics of drugs. Sparse sampling (allowing for fewer samples to be collected) for population pharmacokinetic analysis was combined with opportunistic sampling (where biological fluid is collected as part of samples for standard analysis already planned in patient care) in order to increase the feasibility of the study.

Furthermore, the dried blood spot with the dried matrix (which requires ten times less volume of biological liquid for pharmacokinetic sampling) is increasingly considered in neonates as a potential alternative to traditional plasma sampling [21].

Finally, the frequent use of population pharmacokinetic analysis techniques in neonates has made it possible to obtain more precise information on the pharmacokinetic parameters of both the study population and a single individual, as well as on intra- and interindividual variability.

These methodologies for conducting drug trials in newborns led to the development of new recommendations based on gestational, postnatal, and postmenstrual age.

Neonatal pharmacology is characterized by variable clinical responses to individual doses of a drug; this phenomenon is related to interindividual pharmacokinetic and pharmacodynamic variability, resulting in limited predictability. Choosing a safe drug and an effective dose depends on the full understanding of drug pharmacokinetics and pharmacodynamics, as well as the clinical characteristics of patients receiving the treatment.

## 2. Pharmacokinetics

For each of the four major pharmacokinetic processes, i.e., drug absorption, distribution, metabolism, and excretion, wide differences have been described in qualitative and quantitative data between neonates and adults or other pediatric groups [22,23,24]. Therefore, for optimal dosing of antibiotics in neonates, these factors should be considered in order to reduce the risk of adverse effects or the lack of efficacy.

A drug can have several formulations and can be administered through various routes. The intravenous route is the most frequently used for the treatment of serious neonatal infections. The route of administration can affect both PK and PD processes, and, therefore, needs to be considered when determining optimized dosing recommendations. Peculiarities of the neonatal gastrointestinal tract like elevated gastric pH, longer gastric emptying time, reduced bowel motility, and bile acid production can influence the absorption of orally administered drugs [25,26].

In countries where healthcare may be difficult to access, intramuscular administration is often applied; the main factors affecting the PK parameters in newborns are a variable decrease in blood flow to the muscles over the first 2–3 weeks of life, a higher proportion of water content, and a ratio to muscle mass to body mass less than adults [25].

The two most important parameters of drug pharmacokinetics are the volume of distribution (Vd) and clearance (CL). The elimination half-life (t_1/2_ = 0.693 × Vd/CL), a PK parameter still frequently used, depends on Vd and CL. This means that a prolonged half-life may be explained by a decreased CL, an increased Vd, or both [27].

Newborns, especially those born prematurely, are subject to dynamic changes in physiological parameters such as cardiac output, renal blood flow, and extracellular fluid. Consequently, drug volume distribution, clearance, and half-life vary from day to day, and for some drugs (e.g., aminoglycosides), therapeutic concentrations are very difficult to obtain and maintain (Table 1) [28,29].

### 2.1. Volume of Distribution (Vd)

The volume of distribution represents the theoretical volume (L/Kg) in which the total drug amount in the body tissue should be evenly distributed to reach the same concentration measured in the plasma.

The Vd is defined as a constant of proportionality, which relates the amount of drug administered and the dose to the measured plasma concentration; this parameter is indispensable for calculating at the first administration of the drug (loading dose) and the dose (D) required to obtain the desired plasma concentration (C):

D (mg) = C (mg/L) × Vd (L Kg − 1) × body weight (Kg)

Being a theoretical parameter, Vd can be considerably greater than the total volume of different body compartments.

In neonates and especially preterm infants, the Vd of water-soluble drugs is generally increased due to the large body water content (87% body water for preterm infants, 77% for term infants, and 55% for adults). Increased water proportion in the neonatal organism results in increased Vd values of hydrophilic drugs (e.g., beta-lactams, aminoglycosides, glycopeptides, oxazolidinones (tedizolid)), and antibacterials of different groups (such as colistimethate sodium, dalbavancin, fosfomycin, telavancin). This leads to higher doses per body weight. Not surprisingly, an inadequately low drug serum trough concentration (Cmin) is observed in over 50% of newborns treated with initial vancomycin doses [30].

Compared to full-term neonates, the body composition of preterm babies is characterized by lower fat mass and higher water content. Fetal pulmonary fluid reabsorption after birth determines an increase in extracellular volume that, consequently, results in an increased diuresis and natriuresis [31].

Pathologic conditions, such as patent ductus arteriosus or renal impairment, may determine a reduction in serum drug peak concentrations (Cmax) due to the increased volumes of distribution. Moreover, the extracorporeal membrane oxygenation needs to be considered as an additional compartment when evaluating the PK of antibiotics; it causes an increase in the Vd of hydrophilic antibiotics, and, due to hemodilution, it results in decreased plasma concentrations [32].

Although adipose tissue and skeletal muscle mass are limited in the neonate, a high Vd has also been reported for lipophilic drugs (fluoroquinolones, macrolides and ketolides, metronidazole, rifampicin, trimethoprim/sulfamethoxazole, linezolid (moderate lipophilic)). This appears to be a result of the increased size of lipid-rich organs (e.g., brain and liver) in the newborn compared to their total body weight [25].

The Vd may vary not only according to changes in body composition but also variation in plasma protein binding. The differences described in serum or plasma protein binding might have clinical relevance. Indeed, only the free fraction of antibiotics is able to penetrate the extravascular space where most infections are located, whereas unbound antibiotics are considered to be the pharmacologically active fractions [33,34]. For example, the activities of β-lactam antibiotics have been classified as being mostly dependent on the time that the free (f) concentration is above the minimum inhibitory concentration (MIC) of the pathogen (fT > MIC). The fT > MIC is one of the pharmacokinetic/pharmacodynamic (PK/PD) indices that describe the antimicrobial effect. Importantly, all these indices are based on free concentrations (i.e., the fraction of the total concentration that is not bound to proteins). This finding underlines the importance of protein binding in determining antimicrobial efficacy; an increase or decrease in the plasma protein-binding rate may affect the clinical response while using highly protein-bound drugs (≥90%). Among penicillins, relatively high plasma protein binding rates were reported for cloxacillin (≥94%), dicloxacillin (96–97%), flucloxacillin (95–96%), and oxacillin (92–96%) [25].

For many drugs, the binding to albumin and alpha1-acid glycoprotein is significantly lower in neonates compared to adults. The plasma concentrations of human serum albumin and alpha 1-acid glycoprotein are lower than in adults, resulting in a potential increase in the unbound fraction. The reasons for this bond reduction are various [35]:

(a) albumin and alpha-1 acid glycoprotein concentrations are lower at birth and gradually increase to adult levels by 1 year of age;
(b) some drugs may have a lower binding affinity to fetal albumin that may persist in neonates;
(c) high bilirubin and free fatty acid concentrations can displace drugs from albumin-binding sites;
(d) specific interactions between albumin and globulins can affect the albumin binding affinity.

In summary, body composition, protein concentration, and free water volume changes during the neonatal period contribute to the rapid alteration of antibiotic Vd.

### 2.2. Clearance (CL)

CL is a measure of drug elimination and represents the volume of blood or plasma completely cleared of a certain drug per unit of time (hours or minutes).

This pharmacokinetic parameter allows us to establish the amount of drug to be administered (R_0_ = D·F/τ, where D = maintenance dose; F = fraction of the dose that is absorbed; τ = dose interval) to achieve a desired concentration during a certain dosing interval.

R_0_ = desired concentration × CL;

CL is the result of metabolic and/or excretory processes;

CL = CLm + CLr + CLother;

Clm = metabolic clearance; Clr = renal clearance; CLother = clearance through other processes of elimination.

Both renal and hepatic clearance mature over time, but even full-term infants exhibit a relatively slower clearance value (normalized for body size) than adults. These factors, related to gestational and chronological age, should be considered when developing recommendations on the dosage of antibiotics for newborns [36].

Most of the microsomal enzyme systems responsible for the metabolism of drugs are present at birth, and their activities increase with advancing postnatal and gestational age. Predicting the exact nature of these variations requires knowledge of postnatal maturation and the main enzymes involved. Some paths of biotransformation, including hydroxylation by the P450 monooxygenase system and glucuronidation, demonstrate limited activity at birth.

The ability of the neonatal liver to metabolize drugs is influenced by the ontogeny of many drug-metabolizing enzymes. The frequency of changes in the expression of an enzyme can vary significantly from one individual to another, and they are not always related to changes in other enzymes. For the cytochrome P450 (CYP) enzymes involved in Phase 1 metabolism, each isoenzyme displays its own expression and activity ontogeny profile. At birth, CYP3A7 is the most abundant CYP iso-enzyme, but during the first year of life, CYP3A7 activity decreases, while CYP3A4 (the major iso-enzyme for drug metabolism in adults) displays increased activity during early life. This maturation of the CYP in neonates depends on both postnatal (PNA) and postmenstrual age (PMA) [37,38,39,40].

Drug metabolism can occur in the kidneys, gastrointestinal tract, blood cells, and lungs; however, most drugs are metabolized in the liver, while the elimination of most antibiotics occurs mainly through the renal system [25,41]

For drugs that are really excreted, renal clearance is the net result of glomerular filtration (GFR), tubular secretion, and tubular reabsorption. Compared to full-term neonates and older children, renal function is reduced in the preterm infant due to the immaturity of both glomerular filtration and tubular secretion. The ontogeny of glomerular and tubular functions is driven by age (gestational age and PNA), whereas PNA also causes hemodynamic changes in cardiac output and regional perfusion; the renal clearance of drugs increases with increasing gestational age, PNA, and body weight. [41,42].

Term newborns experience a rapid increase in GFR during the first 2 weeks of life, followed by a steady increase to adult values by 6–12 months of age (2). Premature infants show similar trends, with an initial increase in the GFR that is less steep since nephrogenesis is not completed until 34 weeks of gestation. Although GFR is related to gestational age, this relationship is nonlinear [28,43].

Active tubular secretion and reabsorption are also immature at birth and represent about 20–30% of adult values. Maturation of the active tubular function takes place gradually, reaching adult values within 7–12 months of life. The maturation of tubular reabsorption continues slowly into adolescence, with the steepest increase occurring between 1 and 3 years of age. The drug elimination by these processes depends on the renal blood flow, which increases over time with GFR. In newborns, a reduced binding to proteins will increase the clearance of drugs subjected to these renal processes due to higher concentrations of available unbound drugs [26,43,44].

## 3. Pharmacodynamics

Understanding the PK of antibiotics is necessary but not sufficient for optimizing and individualizing dosing strategies. It is essential to understand the characteristics and dynamics of the target (pathogen) as well [45]. Pharmacodynamic properties of antibiotics are largely similar in different age groups; it is likely that these similarities can be applied to manage neonatal infections [24].

Pharmacodynamics describes the relationship between drug concentration and the pharmacological effect, transforming them into mathematical functions.

**Figure 1:**
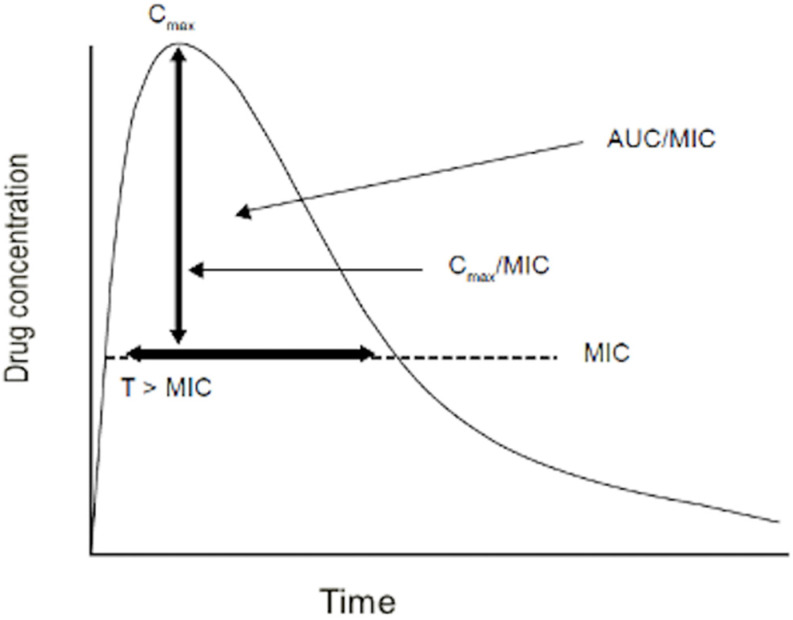
Pharmacodynamic parameters. AUC/MIC: ratio of the area under the plasma drug concentration-time curve (during the interval dosage, e.g., 24 h) to the MIC; Cmax/MIC: ratio of peak plasma drug concentration and the MIC; T > MIC (%): the number of hours or percentage of time the drug plasma concentration remains above the MIC during a dosing interval; Cmax: peak plasma concentration; MIC minimum inhibitory concentration.

The pharmacodynamic and microbiological aspects focus on the effects that the drug exerts on the organism and the pathogen. The management of anti-infective drugs requires the consideration of an additional factor: the pathogen. In other words, the same drug concentration can be effective or sub-therapeutic, depending on the in vitro susceptibility of the pathogen to a given drug (a minimum concentration of the drug that inhibits the pathogen). Currently, the approach based on MIC is the one most frequently applied to correlate drug exposure to microbiological response (Figure 1) (Table 2) [46].

The key pharmacodynamic parameters used to correlate systemic drug exposure to microbiological effects (and thus guide dosing strategies) include

(1) the ratio of the area under the plasma drug concentration-time curve (during the interval dosage, e.g., 24 h) to the MIC (AUC/MIC);
(2) the ratio of peak plasma drug concentration and the MIC (Cmax/MIC);
(3) the number of hours or percentage of time the drug plasma concentration remains above the MIC during a dosing interval (T > MIC (%)) (Figure 1).

The integrated concentration-dependent drug-specific PK/PD marker is the ratio between Cmax/MIC. Aminoglycosides show a concentration-dependent bactericidal effect. Aminoglycoside administration at extended intervals was initially studied in infants at the beginning of the 1990s, but only recently gained wide consensus. Their effectiveness is determined by the relationship between the maximum plasma concentration and the MICs. Therefore, higher concentrations result in faster bacterial elimination. The pharmacodynamics of aminoglycosides are maximized by higher doses administered less frequently. This allows an adequate time for drug renal clearance, thereby minimizing the risk of toxicity.

Unlike aminoglycosides, the specific marker for time-dependent agents (e.g., β-lactams) is the length of time (T) during which concentrations exceed the MIC (T > MIC). The percentage of T > MIC can be calculated by the following equation:

T > MIC (%) = ln (Dose/(Vd × MIC) × (t_1/2_/0.693) × (100/τ)

where T > MIC (%) is the percentage of the dosing interval during which the concentration remains above the MIC. The dose is the amount (mg/kg) of each individual dose, Vd is the apparent volume of distribution (L/kg), t_1/2_ is the half-life (hours), and τ is the dosing interval (hours).

All β-lactams, including ampicillin and semisynthetic penicillin, show time-dependent bactericidal activity. Their effectiveness against sensitive organisms can be determined using the percentage of time plasma concentration that is maintained above the MIC. Higher concentrations above the MIC do not result in a more rapid killing of the microorganism; the infusion of β-lactams (rather than intermittent dosing) maximizes the time above the MIC and presumably optimizes pharmacodynamic targets [47].

The area under the inhibition curve (AUIC24) describes the amount of antimicrobial drug present systemically over a 24 h period (AUC24) in relation to the MIC of the infectious organism; it is a measure of the in vivo potency of that particular dosing regimen. The AUIC describes the amount of active drug present in an individual patient with a specific infection caused by a given pathogen. It can be predictive of efficacy for antibiotics with both concentration and time-dependent activity.

The pharmacodynamic target of vancomycin requires a calculation of the area under the plasma concentration-time curve (AUC). The AUC must then be divided by the MIC. Target values of the AUC/MIC ratio vary depending on the microorganism; for example, an AUC/MIC ratio ≥400 has been associated with a good response to vancomycin therapy in cases of methicillin-resistant *S. Aureus* and coagulase-negative staphylococci bacteremia. Assuming an MIC value equal to 1 mg/L or less, an AUC/MIC value greater than 400 corresponds to Cmin above 15 μg/mL. However, it is important to point out that because of several eventually present variables, an increase in the MIC or a decrease in the AUC can lead to a reduction in the AUIC [46].

## 4. Precision Dosing and Therapeutic Monitoring

The impact of precision dosing, which could determine major improvements, is becoming increasingly important. This is particularly true for the pediatric and neonatal context, where patients are characterized by physiological changes and pathological conditions, making the drug pharmacokinetic behavior unpredictable and responses to therapy uncertain.

Appropriate neonatal dosing must complement the rapid developmental changes that occur in the neonatal period, as evidenced by covariates affecting the kinetics of the drug. Depending on the drug, these covariates are indicators of size and maturity and may include gestational, postnatal, and postmenstrual age, creatinine, tests of liver function, and so on. Additional variables can also be tested: pathological conditions, associated medications, and cardiovascular and ventilatory support. Diseases such as patent ductus arteriosus and perinatal asphyxia or nephrotoxic drugs can cause decreased blood flow or renal impairment that leads to a reduction in GFR. Therapeutic hypothermia (a neuro-protective treatment that lowers a newborn’s body temperature in order to prevent or minimize brain damage after mild-to-severe perinatal asphyxia due to its effects on the cerebral metabolism) affects cardiac output, renal blood flow, and the whole metabolic activity, leading not only to a reduced function of cytochrome enzymes but also a reduced drug clearance through glomerular filtration.

Population pharmacokinetic methods have become the gold standard for drug evaluation in neonatology (Table 3) [48].

These studies use descriptive equations to explain the relationship between physiology and pharmacokinetics, the interindividual variability in these relationships, and their own residual intra-individual variability. Population pharmacokinetic studies use simultaneous data from multiple subjects in populations with drug concentrations collected at different times. This allows random sparse sampling, which may be desirable in neonatal intensive care units; it may allow a robust analysis describing drug pharmacokinetics [61,62].

Modeling applications of population pharmacokinetics in the pediatric field have expanded significantly over the past decade. The development of pharmacokinetic software population growth also coincided with an increasing number of studies in infants. That can be explained by the fact that such studies can be performed with sparse data, reducing invasiveness and making neonates particularly suitable for this type of study.

By applying population pharmacokinetics via nonlinear mixed effects models, covariates can be identified to determine pharmacokinetic parameters together with their variability. Once the pharmacokinetic parameters (typically clearance and volume of distribution) have been estimated, an initial dosing regimen can be set. The software can simulate the effects of administering different doses, the adoption of different infusion frequencies, and finally, the dose and frequency required to achieve the levels of desired antibiotic concentration.

Precision dosing is possible in neonates through Bayesian analysis methods based on models that exploit PK/PD models, bedside drug testing, and electronic decision support tools [63]. The use of dosing strategies based on the Bayesian approach (maximum posterior, MAP) incorporating population pharmacokinetic models and patient-specific pharmacokinetic covariates allows us to predict drug concentrations and optimal dosage regimens, as well as the probabilities—through a mathematical computer simulation—needed to achieve the reference (target) concentrations in individual patients.

Bayesian models have some characteristics that support their use. (a) They can rely on all available information and (b) the statistical inference based on the Bayesian approach is simpler and more intuitive than the one based on the traditional approach. Because of previous reasons, the Bayesian methods are particularly suitable for decision-making problems [64].

Such methods have proven to be useful in the dosage of several antibiotics in infants, significantly increasing the frequency with which target concentrations were achieved.

If residual variability is adjusted through therapeutic drug monitoring (TDM) associated with Bayesian analysis techniques, dosing schedules extrapolated may be subjected to change. [65,66,67]. In such cases, the contribution of the antibiotic TDM becomes essential to ensure adequate systemic exposure to the drug that maximizes therapeutic benefits while minimizing toxicity or side effects (Table 2). However, it requires expertise in different fields.

TDM is more frequently applied

(a) when there is a weak correlation between dose and concentration and the concentration is more closely related to toxicity (or effect) than dosage;
(b) when the drug has a narrow therapeutic index (high risk of toxicity);
(c) when the interindividual variability is elevated; or for some drugs with nonlinear PK.

Variability is known to increase in patients with life-threatening infections. In such cases, rapid pathophysiological fluctuations may occur, even over the course of a few hours; they can influence the pharmacokinetics, and, therefore, the relationship between dose and antibiotic exposure. Reliable measurements are a prerequisite for effective TDM; accordingly, turn-around times >24 h should be disregarded for critically ill patients [44].

It is important to underline that the advanced practice of TDM exploits Bayesian modeling and simulation methods to guarantee a safe and effective antibiotic therapy in newborns based on defined procedures such as the measurement of plasma drug concentrations, the development of population pharmacokinetic models with variability quantification of key pharmacokinetic parameters, such as clearance and Vd, the identification of time-dependent factors influencing key pharmacokinetic parameters during the first weeks of life, and the characterization of aspects of pharmacodynamics and target levels of antibiotics in newborns [68,69]. Therefore, adequate antibiotic drug monitoring requires expertise in different fields and calls for the collaboration of physicians together with lab technicians and quantitative clinical pharmacologists.

The optimal choice of antibiotic treatment (regimen and duration) is an important element in order to reduce antibiotic misuse and overuse among neonates, which are associated with adverse effects, including antibiotic resistance, increased risk of invasive candidiasis, necrotizing enterocolitis, viral and bacterial superinfections, death, and long-term effects, like early childhood obesity and chronic diseases later in life [69,70,71,72,73,74,75,76,77].

Antimicrobial stewardship programs (ASP) consist of a set of strategies that aim to improve the appropriateness of antimicrobial prescription and minimize adverse effects; the aim is achieved through the selection of the optimal antibiotic regimen, dose, duration, and route of administration and is based on a multidisciplinary approach [78,79]. Therefore, pharmacists (hospital and/or clinical) can be involved in ASPs to facilitate appropriate antibiotic use. A recent systematic review on this topic showed that involving pharmacists in ASPs reduces both the use and duration of antibiotic treatment in critically ill neonates [80,81].

## Figures and Tables

**Table 1:** Summary of the pharmacokinetic parameters of gentamicin, tobramycin, amikacin, and netilmicin [28].

Population Averages (±SD) in Patients with Normal Renal Function.					
Parameter	Children	Neonates			
		<2000 g		>2000 g	
		<1 Week	>1 Week	<1 Week	>1 Week
Clearance mL/min/1.73 m^2^	1.31 ± 10	22.1	24.6	28.4	36.4
Volume of distribution, L/kg	0.07–0.7	0.2–0.7			
Elimination half-life, h	0.5–2.5	2.0–9.0			
% excreted in urine	85–95%	First dose 65–85% in 24 h; steady-state 85–95%			

**Table 2:** Pharmacokinetic and pharmacodynamic targets for antibiotics. Adapted from [26].

	Aminoglycosides			β-lactams			Glycopeptides
	Gentamicin	Amikacin	Tobramycin	Penicillins	Carbapenems	Cephalosporins	Vancomycin
PK/PD parameters	Cmax/MIC			fT > MIC (%)			24 h AUC/MIC
PK/PD efficacy target values	≥8	8–10	8–12	T > MIC >50–60%	T > MIC >40–60%	T > MIC >60–70%	400
TDM reference intervals	Cmin 0.5–2.0 mg/L	Cmin <5 mg/L	Cmin <1.0 mg/L				Cmin 10–20 mg/L
TDM reference intervals	Cmin 0.5–2.0 mg/L	Cmin <5 mg/L	Cmin <1.0 mg/L				Cmin 10–20 mg/L
	Cmax 5–10 mg/L	Cmax 20–60 mg/L	Cmax >10 mg/L				Cmax 30–40 mg/L
PK/PD properties	Concentration-dependent killing and prolonged persistent effects			Time-dependent killing and minimal persistent effects			Time- and conc.-dependent killing and moderate to prolonged persistent effects

Cmax: peak plasma concentration, Cmin: trough plasma concentration, MIC: minimum inhibitory concentration, AUC: area under the concentration-time curve, 24 h AUC: area under the concentration-time curve over 24 h, fT > MIC: percentage of time for which the free fraction of drug remains above MIC, TDM: therapeutic drug monitoring.

**Table 3:** Covariates used in some studies by applying a population pharmacokinetic model in newborns.

Drug	No. of Pts	PN Age Days	Software	Covariables Used to Estimate Clearance	Covariables Used to Estimate Volume of Distribution	Bibliography
Amikacin	131	1	NPEM2	GA, Weight	GA, Weight	[49]
Amoxicillin	150	1	MW/PHARM	GA, PNA, Weight	Weight	[50]
Cefepime	55	14.5	NONMEM	SCr	PCA	[51]
Cefepime	31	21.8	NONMEM	ASC, CLcr	ASC	[52]
Ceftixozime	50	5	NONMEM	Weight	Weight	[53]
Gentamicin	30	7	NONMEM	Weight	Weight	[54]
Gentamicin	79	4.2	NONMEM	Weight, GA	Weight	[55]
Gentamicin	200	5.5	NONMEM	Weight, Clcr, PNA	Weight	[56]
Gentamicin	61	20	NONMEM	Weight, GA, PNA	Weight, GA	[57]
Meropenem	37	40	NONMEM	Scr, PCA	Weight	[58]
Vancomycin	49	2.3	NONMEM	PNA, Weight	Weight	[59]
Vancomycin	214	12	NONMEM	Weight, PMA	Weight	[60]

GA: gestational age; PNA: postnatal age; PCA: postconceptional age; PMA: postmenstrual age; BSA: body surface area; Scr: serum creatinine; Clcr: creatinine clearance.
